# Ammonia oxidation by aerobic methanotrophs as a source of marine nitrous oxide

**DOI:** 10.1093/ismejo/wraf242

**Published:** 2025-10-31

**Authors:** Sai Yang, Jiawei Zhang, Yafei Ou, Wenxiao Liu, Xinru Tian, Li-Jun Hou, Hong-Po Dong

**Affiliations:** State Key Laboratory of Estuarine and Coastal Research, East China Normal University, Shanghai 200241, China; State Key Laboratory of Estuarine and Coastal Research, East China Normal University, Shanghai 200241, China; State Key Laboratory of Estuarine and Coastal Research, East China Normal University, Shanghai 200241, China; State Key Laboratory of Estuarine and Coastal Research, East China Normal University, Shanghai 200241, China; State Key Laboratory of Estuarine and Coastal Research, East China Normal University, Shanghai 200241, China; State Key Laboratory of Estuarine and Coastal Research, East China Normal University, Shanghai 200241, China; State Key Laboratory of Estuarine and Coastal Research, East China Normal University, Shanghai 200241, China

**Keywords:** methanotrophs, ammonia oxidation, nitrous oxide

## Abstract

Aerobic methanotrophs encode a hydroxylamine oxidoreductase, which facilitates the oxidation of ammonia to nitrite or nitric oxide, potentially leading to nitrous oxide production. Aerobic methane oxidation has been documented in shallow marine waters or the water column of the open ocean. However, little is known about the distribution pattern of marine aerobic methanotrophs containing hydroxylamine oxidoreductase and their contribution to marine nitrous oxide emissions. Here, by analyzing global marine metagenomes, we show that hydroxylamine oxidoreductase-containing aerobic methanotrophs were widely distributed across diverse marine habitats, with higher abundances in methane seep systems and estuary regions than in other environments. Among these, aerobic methanotrophs belonging to *Gammaproteobacteria* were the most widely distributed and abundant functional group. We also identified a second order within *Gammaproteobacteria* (Ga0077536) potentially capable of aerobic methanotrophy, and a complete repertoire of denitrification genes in a gammaproteobacterial methanotroph, expanding the phylogenetic and functional diversity of marine aerobic methanotrophs. By using enrichments of estuarine methanotrophs in combination with ^15^N stable isotope tracing and metatranscriptomic analysis, we indicate that marine aerobic methanotrophs take part in ammonia oxidation and nitrous oxide production. The ammonia oxidation can persist for ~6 days, and the nitrous oxide produced is at least partially derived from the hydroxylamine oxidation. Given the prevalence of denitrification genes in aerobic methanotrophs, methane oxidation may also be coupled to NO_x_^−^ reduction under anoxic marine conditions, potentially contributing to nitrous oxide production. The intrinsic nature of aerobic methanotrophs could partially offset the mitigation of global warming achieved through the methane consumption.

## Introduction

Aerobic methanotrophs (MOB) oxidize methane (CH_4_) to methanol through a particulate (pMMO) or soluble CH_4_ monooxygenase (sMMO) using oxygen (O_2_). Among these, pMMO—encoded by the *pmoCAB* operon—is the enzyme most commonly utilized by MOB for CH_4_ oxidation [1]. Owing to their prevalence and functional significance, *pmo* genes also serve as important molecular markers for assessing methanotroph diversity and identifying lineages previously uncharacterized in natural environments [1]. To date, aerobic methanotrophs were only found in four bacterial phyla: *Pseudomonadota*, *Verrucomicrobiota*, *Gemmatimonadota*, and *Actinomycetota* [2, 3]. With the exception of *Gemmatimonadota*, which is identified based on the presence of *pmo* genes in its genome [4], representatives of these phyla have been isolated. The rapid expansion of marine metagenomic databases has made it possible to discover new lineages of MOB from marine environments.

MOB are generally believed to exclusively inhabit oxic environments because O_2_ is required for the activation of pMMO or sMMO. However, recent studies have frequently detected them in various anoxic habitats, such as the water columns and sediments of lakes and oceans [5–7]. It has been suggested that these aerobic methanotrophs may couple CH_4_ oxidation with anaerobic respiration processes, such as reduction of nitrate (NO_3_^−^), nitrite (NO_2_^−^), nitrous oxide (N_2_O), and iron, or with fermentation, to overcome O_2_ limitation [2, 8]. These anoxic waters and sediments are often abundant in ammonia (NH_3_) due to the rapid mineralization of labile organic matter [9]. When CH_4_ is depleted, aerobic methanotrophs in NH_3_-rich environments may oxidize NH_3_ to hydroxylamine (NH_2_OH), despite lacking the canonical NH_3_ monooxygenase [10, 11]. The reaction is catalyzed by pMMO in MOB and is attributed to the structural similarity between CH_4_ and NH_3_ [12]. In MOB strains capable of oxidizing NH_3_, a hydroxylamine oxidoreductase-like enzyme (mHAO), homologous to that found in ammonia-oxidizing bacteria (AOB), has been identified [10]. Recently, a HAO protein was purified from the verrucomicrobial thermoacidiphilic methanotroph *Methylacidiphilum fumariolicum*, which is capable of converting NH_2_OH to nitric oxide (NO) [11]. NH_2_OH, structurally similar to methanol, can compete with methanol for binding to the active site of methanol dehydrogenase [11, 13]. The presence of mHAO helps mitigate the inhibitory effect of NH_2_OH on methanol dehydrogenases that are required for oxidation of methanol, thereby enabling methanotrophs to thrive in CH_4_- and NH_3_-rich habitats [11]. Nevertheless, the reaction catalyzed by the mHAO facilitates the production of NO and NO_2_^−^, which can be further converted to N_2_O by NO reductase and NO_2_^−^ reductase under anoxic conditions. A few of cultured methanotrophs have been reported to produce N_2_O during NH_3_ oxidation [10, 14].

To date, physiological and metabolic features of marine aerobic methanotrophs are poorly understood although aerobic CH_4_ oxidation rates have been broadly detected in the water columns of the different open oceans and shallow coastal waters [15–17]. Recently, high CH_4_ oxidation rates have been observed in coastal waters of East China Sea, which are correlated with methanotrophic biomass [18]. It is estimated that coastal aerobic methanotrophs annually consume roughly half of the CH_4_ produced in near-shore waters (< 50 m), amounting to 1.8 ± 2.7 Tg [18]. In addition, marine cold seep systems and the overlying water columns are also hotspots for aerobic CH_4_ oxidation [19]. If we hypothesize that mHAO-containing aerobic methanotrophs in CH_4_- and NH_3_-rich marine habitats contribute to NH_3_ oxidation, the process may be a yet unrecognized source of marine N_2_O.

Here we investigate the distribution patterns of mHAO-containing aerobic methanotrophs across global oceans and analyzed their metabolic potentials. Furthermore, we obtained the MOB enrichment cultures from estuarine water and sediment samples, in which no canonical NH_3_ oxidizers were detected. Upon adding NH_3_ under CH_4_-free conditions, we observed N_2_O production in these enrichments where NH_3_ oxidation occurred. Stable isotope tracing experiments and transcriptomic analyses further revealed that NH_2_OH oxidation by MOB contributed partially to N_2_O production. We suggest that mHAO-containing aerobic methanotrophs are widely distributed in marine environments and may be a new source of oceanic N_2_O.

## Materials and methods

### Retrieval of *mhao*-like gene fragments in marine metagenomes

To assess the abundance of *mhaoA* sequences in global marine metagenomes, we constructed a custom database containing HAO subunit A sequences from aerobic CH_4_-oxidizing bacteria, AOB, complete ammonia oxidizer (comammox) *Nitrospira*, and anaerobic ammonium oxidation (anammox) bacteria (Supplementary Table 1). Inclusion of nonmethanotrophic sequences aims to introduce competitive recruitment for reads, thereby minimizing the occurrence of false positive hits. The HaoA sequences in the custom database were retrieved by annotating bacterial protein sequences from Genome Taxonomy Database (GTDB, release 220, https://data.ace.uq.edu.au/public/gtdb/data/releases/release220/) using HMMER (v3.4) [20] and BLASTP (v2.9.0+) [21]. Taxonomy of the resulting sequences were confirmed by inferring gene trees. A total of 2175 metagenomic datasets from global oceans were downloaded from the NCBI Sequence Read Archive (SRA, https://www.ncbi.nlm.nih.gov/sra). Information for sampling sites including habitats, locations, and depths, is summarized in Supplementary Tables 2 and 3. These SRA files were converted to FASTQ files using Fastq-dump using —spilt-3 (https://github.com/ncbi/sra-tools/tree/master/test/external/fasterq-dump) and then processed with Sickle (v1.33) [22]. The reads in metagenomes were identified by searching against the above custom database using DIAMOND's BLASTX (*32*) (v0.9.25) (cutoff: e-value <1e-5, identity >75%, alignment length > 50%). To compare the abundance of *haoA* genes across different metagenomes, the counts per million (CPM) method was used [23].


(1) \begin{equation*} \mathrm{CPM}=\frac{M}{T}\times{10}^6 \end{equation*}

where *M* is the number of reads mapped to the *haoA* genes, and *T* is the number of total reads in a metagenomic sample.

### Genome assembly, binning, annotation, and phylogeny

Only a subset of metagenomes with relatively high *mhaoA* abundance was selected for *de novo* assembly and genome binning, as metagenome-assembled genomes (MAGs) harboring *mhaoA* gene are more readily recovered from these datasets. The raw reads in metagenomes were quality controlled to obtain clean data using Trimmomatic software (v0.38) [24]. The resulting clean reads were then assembled into contigs/scaffolds using MEGAHIT (v1.2.9) [25] (--presets meta-large, --min-contig-len 500) or IDBA-UD [26] (-mink 34, -maxk 124, -steps 10, --min_contig 500, --pre_correction). The assembled sequences were binned using MetaBat2 (v2.12.1) [27] eight times, with eight combinations of specificity and sensitivity parameters (--maxP 95 or 60, --minS 60 or 95; --maxEdges 200 or 500). The resulting bins were refined with DAS Tool (v1.1.4) [28] (−-score threshold 0.25). Abnormal or contaminating contigs/scaffolds were removed using mmgenome [29] and RefineM (v.0.0.14) [30]. Completeness, contamination, and strain heterogeneity of bins were evaluated with CheckM2 (v1.1.0) [31]. Taxonomic information of bins was obtained using the GTDB Toolkit (v 2.2.6) [32] (Supplementary Table 4). These genomes containing *pmo* genes or belonging to known lineages of aerobic methanotrophs were selected and their species tree was constructed using IQ-TREE (v1.6.12) [33], based on a concatenated set of 120 bacterial-specific marker genes from GTDB.

Open reading frames in MAGs were predicted using Prodigal (v2.6.3) with the parameter -p meta [34]. Gene annotation was obtained by searching against nonredundant database using BLASTP (v2.9.0+) (e-value <1e-5) [21]. Interest genes were confirmed by comparison with the pfam database using HMMER (v3.4) [20]. Gene trees were computed with IQ-TREE (v1.6.12) [33] using the command: “-m TEST (GTR+F+I+G4) -bb 1000.”

### Sample collection

The Yangtze Estuary is located at the mouth of the Yangtze River (Supplementary Fig. 1). Human activities have led to eutrophication of the Yangtze River Estuary and its adjacent regions. High NH_4_^+^ and CH_4_ concentrations were frequently detected in the estuary [18, 35]. Therefore, the Yangtze River estuary is an ideal place to study the role of aerobic methanotrophs in NH_3_ oxidation.

Surface water and sediment samples were collected from two sites (ST1 and ST2) in the Yangtze Estuary (Supplementary Fig. 1) onboard the Research Vessels “Kexue 3” during expeditions in June and December of 2023. Seawater samples were collected using Niskin-X bottles equipped with a conductivity-temperature-depth profiler (Sea-Bird 911 plus). A 2 l seawater aliquot was immediately filtered through a 0.2 μm sterile PC membrane (Milipore). The filters were stored at −20°C for subsequent DNA extraction, and the filtrates were stored at 4°C for later nutrient analysis. Another 2 l seawater aliquot was kept in the dark at 4°C for subsequent culture experiments. Subtidal sediments were collected using a box core sampler onboard. The surface layer (0–2 cm) of the sediment core was sliced and placed into sterile bags. A portion of the subsamples was stored at 4°C for subsequent cultivation experiments, while the remainder was kept at −20°C for DNA extraction.

### Enrichment experiments supporting NH_3_ oxidation and N_2_O production by aerobic methanotrophs

Surface water and sediment samples from two sites (ST1 and ST2) of the Yangtze River Estuary were incubated in 1 l sealed glass bottles, each equipped with two sampling valves. The cultures were maintained in the dark at 25°C with a shaking speed of 200 rpm for a period of 60 days. To support microbial growth, the nitrate mineral salts (NMS) medium [36] was used (see Supplementary materials).

For microbial cultivation of water samples, 500 ml of seawater was directly supplemented with the components of the NMS medium. For sediment samples, 60 g of fresh sediment was mixed with 440 ml of the NMS medium to form a slurry. At the beginning of the enrichment process, high-purity CH_4_ gas is injected into the headspace, raising the CH_4_ concentration in the headspace to ~20% (v/v). CH_4_ concentrations in the headspace were regularly monitored using a gas chromatograph (GC-2014, Shimadzu, Kyoto, Japan). When the CH_4_ concentration dropped below 1.9 ppm, a new round of CH_4_ injection was carried out. Every 15 days, half of the cultures in the bottles were replaced with fresh NMS medium to promote the growth of MOB. This process was repeated three times. After 45 days of incubation, the enriched cultures were transferred to new bottles and diluted with fresh NMS medium at a 1:50 ratio. In addition, 50 μM of phenylacetylene was added to these cultures to inhibit the growth of ammonia-oxidizing archaea (AOA) and AOB [37, 38]. During the enrichment period, the concentrations of NH_4_^+^ and NO_2_^−^ were monitored at regular intervals. The abundances of MOB, NH_3_ oxidizers, and total bacteria were determined every two weeks based on quantitative PCR of the *pmoA* gene of MOB [39], the *amoA* genes of AOB [40], AOA [41], and comammox [42, 43], as well as the bacterial 16S rRNA genes [44] (Supplementary Table 5). By Day 60, the *amoA* gene of canonical NH_3_ oxidizers was no longer detectable by either agarose gel electrophoresis or gene quantification (Supplementary Figs. 2 and 3).

On Day 60, the enrichment experiment was completed, and the enriched cultures were used to investigate the effects of varying CH_4_ concentrations on NH_3_ oxidation rates (AORs) and N_2_O production rates (N_2_OPRs) by MOB. The MOB enrichment was mixed with fresh NMS medium at a 1:1 ratio and transferred into 120 ml serum bottles. High-purity CH_4_ gas is then injected into the headspace of the bottles, setting four CH_4_ concentration gradients: 0.05%, 0.1%, 1%, and 5% (v/v). Once equilibrium was reached between the gas and liquid phases, the CH_4_ concentration in the headspace was measured using a gas chromatograph. The corresponding partial pressures of CH_4_ at these concentrations were 54, 109, 1099, and 5495 Pa, respectively. Three replicates were set up for each concentration gradient.

### Measurement of NH_3_ oxidation and N_2_O production rates

The NH_3_ oxidation rate was measured following methods outlined in previous studies [45, 46], where the δ^15^N in NO_X_^−^ (NO_2_^−^ + NO_3_^−^) was determined with the denitrifier method. The N_2_O production rate was measured following our previous method with minor modifications [47]. Detailed protocols are provided in Supplementary materials.

### DNA extraction, 16S rRNA gene amplicon sequencing, and OTU analysis

Total DNA from *in situ* samples, as well as enriched water and sediment samples, was extracted using the DNeasy PowerSoil Pro Kit (QIAGEN, USA) following the kit’s instructions. DNA concentration and purity were measured using a NanoDrop-2000 spectrophotometer (Thermo Scientific, USA). The 16S rRNA genes from *in situ* samples and samples enriched for 60 days were amplified using a two-step PCR procedure for barcoded pyrosequencing with the primer pair 515F and 806R [48, 49] (Supplementary Table 5). The target gene bands were then recovered and purified, followed by sequencing on a Hiseq 2000 sequencer in Shanghai Biotechnology Corporation. The reads were subsequently merged using the PEAR software (v0.9.8) [50]. Sequencing of the 16S rRNA genes from eight samples yielded a total of 782 013 reads after quality control. To eliminate nontarget gene sequences, 16S rRNA gene sequences were filtered based on the SILVA rRNA database (v138) to remove nonbacterial and nonarchaeal sequences [51]. The remaining sequences were clustered into operational taxonomic units (OTUs) using the Usearch (v11.0.667) [52, 53], based on a 97% similarity cutoff. More detailed procedures can be found in Supplementary materials.

### Metagenomic and metatranscriptomic analyses of the enrichment cultures

The MOB enrichment cultures derived from ST2 water samples were centrifuged at 12 000 g for 10 min at 4°C. The resulting pellets were then washed once with NMS media without NO_3_^−^ and centrifuged again. One portion of the pellets was used for metagenomic sequencing. Genomic DNA was extracted using a commercial DNA extraction kit (MoBio Laboratories, Carlsbad, USA). Metagenomic sequencing was performed on the HiSeq 2500 System (Illumina) at Novogene Co., Ltd (Beijing, China), generating ~40 Gbp of paired-end reads (2 × 150 bp) for the enrichment sample. Genome assembly and binning of the metagenomic data were performed as described above. The relative abundance of MAGs was calculated as the percentage of mapped reads relative to the total number of clean reads.

For metatranscriptomic samples, another portion of the pellets were suspended in NMS containing NH_4_Cl (1 μM ^15^NH_4_Cl and 10 μM nonlabeled ^14^NH_4_Cl, final concentration), excluding NO_3_^−^, and incubated for 6 days. Samples were collected at 0, 12, and 24 h for metatranscriptomic analysis, with three biological replicates prepared for each time point. RNA sequencing was performed using the NovaSeq System (Illumina) at GENWIZ (Suzhou, China). Bin132, recovered from the ST2 metagenome and affiliated with the genus *Methylotuvimicrobium*, was used as the reference genome for metatranscriptomic analysis. Clean metatranscriptomic reads were mapped to the genes of bin132 using Kallisto (v0.46.0) [54] with default parameters to estimate transcript abundance. Gene expression levels were quantified as Transcripts Per Million (TPM). Differences in gene expression across time points were evaluated using two-tailed paired t-tests. More detailed information is provided in Supplementary materials.

### *SP* analysis of N_2_O produced from MOB enrichments

To identify the sources of N_2_O production in the enrichment cultures, we performed natural isotopic analysis of N_2_O. Briefly, 20 ml of the MOB enrichment cultures were transferred to 120 ml serum bottles and aerated using high-purity air. The cultures were supplemented with nonlabeled ^14^NH_4_Cl (final concentration: 10 μM) before being sealed with butyl rubber stoppers and aluminum caps. Following a 48-h incubation period, headspace gas was collected and analyzed using isotope ratio mass spectrometry (IRMS, Delta V Plus, Thermo Fisher Scientific, Bremen, Germany). The calculation methods for δ^15^N^β^ and site preference (*SP*) are described in Supplementary materials.

## Results and discussion

### mHAO-containing methanotrophs are widely distributed throughout the global oceans

**Figure 1:**
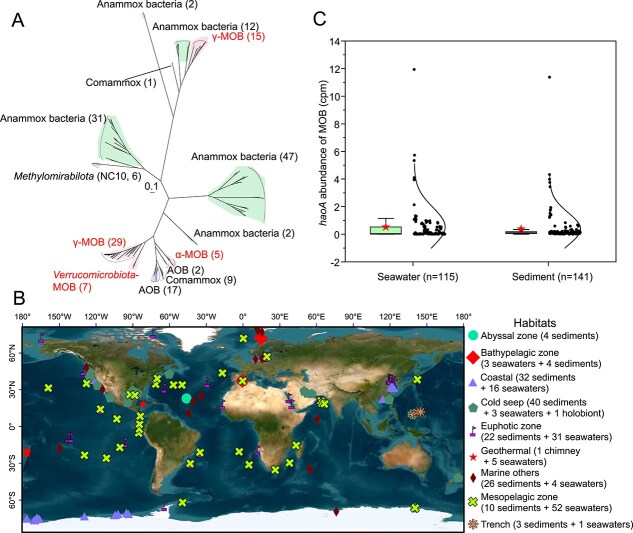
Phylogeny of mHAO subunit A sequences of MOB and their gene (*haoA*) distribution in global oceans. (A)Phylogenetic analysis of mHAOs subunit A sequences in bacteria. The maximum-likelihood tree was inferred using IQ-TREE (LG + I + G4, −bb 1000). The number of sequences for branches is given in parenthesis. The scale bar indicates 0.1 amino acid substitutions per site. The sequences used to construct the tree are listed in Supplementary Table 1. AOB, ammonia-oxidizing bacteria; Anammox bacteria, anaerobic ammonium oxidation bacteria; Comammox, complete ammonia oxidizer. (B) Sampling locations of 258 metagenomic samples. Their habitat types are marked by different icons. Source: Esri, Maxar, Earthstar Geographics, and the GIS user community. (C) The abundance of MOB *haoA* in seawater and sediment samples. Average, median, and interquartile rang are shown.

Many methanotrophs encode mHAO in their genomes to eliminate NH_2_OH, which significantly suppresses calcium-dependent and lanthanide-dependent methanol dehydrogenases [11]. Of the 34 isolated species of MOB, 28 were found to possess the gene encoding mHAO (Supplementary Table 6). Phylogenetic analysis revealed that mHAO subunit A sequences from MOB were divergent from those from AOB, comammox *Nitrospira*, and anammox bacteria (Fig. 1A). The result indicates that the gene encoding the mHAO alpha subunit (*mhaoA*) can be used to track the abundance of *mhaoA*-containing MOB in environmental samples.

We compiled 2175 metagenomic samples from various regions of the global ocean. Reads related to *mhaoA* in these metagenomes were identified by comparison with a custom database containing HAO subunit A sequences from MOB, AOB, comammox *Nitrospira*, and anammox bacteria. Addition of nonmethanotrophic sequences aims to allow them compete with mHAOA so that reads belonging to *mhaoA* can be more accurately identified. Of these metagenomes, 258 were found to harbor *mhaoA-*like sequences, with 143 originating from sediments and 115 from seawater samples. These samples were primarily from the Pacific Ocean, Atlantic Ocean, Arctic Ocean, South China Sea, Ross Sea, Amundsen Sea, Baltic Sea, Bohai Sea, Yangtze estuary, and other regions, which covered diverse marine habitats including coastal regions, epipelagic and mesopelagic zones of the ocean, deep-sea hydrothermal vents, cold seep environments, abyssal zones, and oceanic trenches (Fig. 1B, Supplementary Tables 2 and 3).

The *mhaoA-*like sequences were detected in metagenomic samples collected from 48 layers of the ocean’s water column, spanning depths from 2 m to 5000 m (Supplementary Table 2). Their abundance varied from 0.003 to 11.933 CPM (*mhaoA-*like sequences per million sequencing reads Fig. 1C), with notable peaks observed at depths of 40 m, 375 m, 1127 m, and 2107 m (Supplementary Table 2). Three samples from deep-sea cold seep water in South China Sea (depths of 1120–1127 m) were found to contain higher abundance of *mhaoA-*like sequences (Supplementary Table 2), suggesting that activity and size of MOB community are determined by availability of CH_4_. Moreover, a high abundance of *mhaoA-*like sequences was identified in several samples from chlorophyll maximum layers and mesopelagic zones of the ocean, implicating that the environmental factors controlling the activity of methanotrophic community are complex. In addition to concentrations of CH_4_ and O_2_, the abundance of trace metals and the physical transport of water masses may also influence the distribution of MOB [19]. A substantial proportion of *mhaoA*-like sequences was detected in 16 water samples collected from the Yangtze River Estuary, with the highest abundance observed at station ST1. This may help explain high aerobic CH_4_ oxidation rates previously documented in the region [18]. The CH_4_ sources in the estuarine waters of the Yangtze river have been shown to be derived from cleavage of methylated compounds and sediment release [18].

A total of 141 sediment samples were found to harbor *mhaoA-*like sequences, with abundances ranging from 0.002 to 11.373 CPM (Fig. 1C), of which 29.08% were from cold seep environments and 22.7% were from the intertidal or subtidal sediments of the estuary (Supplementary Table 3). The highest abundance value was recorded at cold seep sites near the Southern Hikurangi Margin in New Zealand. Although O_2_ is essential for the initial step of CH_4_ oxidation by MOB, their activity and growth are often observed in hypoxic or anoxic habitats such as stratified water column or sediments [5, 6]. It has been suggested that in these MOB, CH_4_ oxidation may be coupled with iron reduction, denitrification, or fermentation.

Based on GTDB taxonomy, *mhaoA* can be assigned to the classes *Gammaproteobacteria* and *Alphaproteobacteria*, as well as to the phylum *Verrucomicrobiota*. Among these three types, the *mhaoA* sequences for Gamma-MOB were the most widely distributed (found in 91.41% of metagenomic samples), followed by those for *Alphaproteobacteria* (51.16%) and *Verrucomicrobiota* (18.22%). In the majority of metagenomic samples, Gamma-MOB *mhaoA* sequences were predominant in terms of abundance. The wide distribution and predominance of Gamma-MOB *mhaoA* sequences in marine environments are basically in accordance with the results obtained using *pmoA* or 16S rRNA genes [55, 56]. Although Alpha-MOB *mhaoA* sequences were generally low in abundance, elevated percentages were observed in water samples from the Yangtze estuary and euphotic zones of the Arctic Ocean (Supplementary Table 2), as well as in sediment samples from the Bornholm Basin and cold seep regions of the Atlantic Ocean (Supplementary Table 3), suggesting that specific environmental conditions favor the selection of certain types of methanotrophs. Totally, our findings indicate that mHAO-containing MOB are widely distributed across various marine environments. In environments where CH_4_ and NH_3_ co-occur, the mHAO helps CH_4_-oxidizing bacteria to perform NH_3_ oxidation. Under hypoxic and anoxic conditions, HAO-containing methanotrophs could produce N_2_O from NH_3_-derived NO and NO_2_^−^ [8, 57].

### Genomic analysis of aerobic methanotrophs from marine environments

**Figure 2:**
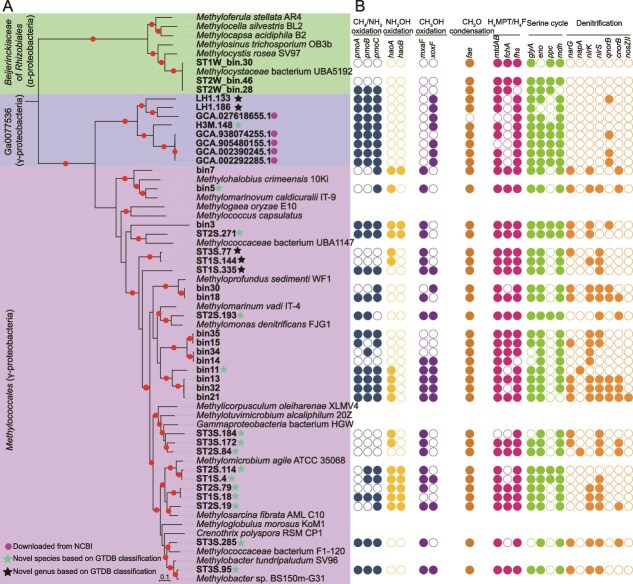
Phylogeny and metabolic potential of MOB MAGs recovered from oceanic metagenomic samples. (A) Maximum-likelihood tree showing relationship between reconstructed bins with reference MOB. The tree was constructed using IQ-TREE (LG + F + I + G4) based on a concatenated set of 120 bacterial-specific marker proteins, rooted with verrucomicrobial methanotrophs. Ultrafast bootstrap value >90% are shown with solid circles. (B) The distribution of key genes related to CH_4_/NH_3_ oxidation and denitrification process. *Pmo*, pMMO; *hao*, NH_2_OH dehydrogenase; *mxaF*, methanol dehydrogenase (cytochrome c) subunit 1; *xoxF*, lanthanide-dependent methanol dehydrogenase; *fae*, 5,6,7,8-tetrahydromethanopterin hydro-lyase; *mtdA*, methylenetetrahydrofolate/methylenetetrahydromethanopterin dehydrogenase (NADP+); *mtdB*, methylene-tetrahydromethanopterin dehydrogenase; *fchA*, methenyltetrahydrofolate cyclohydrolase; *fhs*, formate–tetrahydrofolate ligase; *glyA*, serine hydroxymethyltransferase; *eno*, enolase; *ppc*, phosphoenolpyruvate carboxylase; *mdh*, malate dehydrogenase; *narG*, membrane-bound NO_3_^−^ reductase; *napA*, periplasmic NO_3_^−^ reductase; *nirK*, copper-containing NO_2_^−^ reductase; *nirS*, cytochrome cd1 NO_2_^−^ reductase; *qnorB*, quinol-oxidizing single-subunit class NO reductase; *cnorB*, cytochrome bc-type NO reductase, *nosZ*II, clade II type N_2_O reductase.

We selected 11 metagenomes with relatively high *mhaoA* abundance for *de novo* assembly and genome binning. A total of 34 high- and medium-quality MAGs (completeness >70% and contamination <10%), potentially belonging to MOB, were obtained (Fig. 2 and Supplementary Table 4). Of these MAGs, 18 were derived from coastal sediment samples, 10 from cold seep sediments samples, 3 from estuarine waters, and 3 from hydrothermal vent chimney environments (Supplementary Table 4). Among these, 28 bins affiliated with *Methylomonadaceae*, *Methylothermaceae* or an uncharacterized family DRLZ01 of the *Methylococcales* of *Gammaproteobacteria*, three bins affiliated with *Beijerinckiaceae* of the *Rhizobiales* of *Alphaproteobacteria*, and three bins affiliated with an as yet uncharacterized order Ga0077536 of *Gammaproteobacteria*. Approximately 80% of these bins contained one, two, or all three subunit genes (*pmoABC*) of pMMO, the key enzyme for CH_4_ oxidation of bacteria. For the other bins that lack a pMMO, their closest relatives in the same cluster often contain the enzyme, indicating that its absence may be attributed to genomic incompleteness. The Ga0077536 is a newly discovered order within *Gammaproteobacteria* and its members have not been reported to use CH_4_ as an energy source. Here, we assembled three MAGs (LH1.186, LH1.133, and H3M.148) from metagenomes generated from saltmarsh and mangrove wetlands that belong to the Ga0077536 order. They encode a putative pMMO that forms a distinct group with those of additional five MAGs recovered from subsurface seawater (downloaded from public databases) and branches as a sister lineage with those of the recently discovered *Candidatus* Methylotropicum kingii of Gemmatimonadota [4] (sharing about 47.8%–48.5% sequence identity) (Supplementary Fig. 4A and Table 7). Molecular modeling reveals that the enzyme possesses a conserved tertiary structure and copper-binding residues akin to those found in canonical pMMOs (Supplementary Fig. 4B–D). In addition, similar to other methanotrophs, these eight bins also encode most of the enzymes involved in oxidation of methanol and formaldehyde, as well as those of the tetrahydromethanopterin/tetrahydrofolate pathway, and the serine cycle (Fig. 2B). The results suggest that Ga0077536 is a second order potentially capable of performing aerobic CH_4_ oxidation. Isolating these bacteria, along with related physiological studies, are required to verify their ecological roles.

About half of the MAGs encode one or two subunits of mHAO (Fig. 2B). For the remaining MAGs, it is unclear whether the absence of the *hao* genes is due to incomplete genome assembly or reflects their true absence. It is also likely that a subset of marine aerobic methanotrophs genuinely lack the *hao* genes. We noticed that most of these MAGs contained denitrification genes, including NO_3_^−^ reductases (*narG* or *napA*), NO_2_^−^ reductases (*nirK* or *nirS*), NO reductase (*nor*), and N_2_O reductases (*nosZ*) (Fig. 2B), which are also found in genomes of aerobic methanotrophs from anoxic lakes, wetlands, acidic forest soil, and rice paddy fields [2, 6, 58, 59]. These denitrification genes were mainly distributed in MAGs derived from sediments across diverse marine habitats. Only *no*r genes were detected in three MAGs obtained from subsurface waters of the North Sea and Bohai Bay. To date, none of the known aerobic methanotroph genomes or MAGs encode a complete repertoire of denitrification genes [2]. However, we identified a complete set of denitrification genes in bin21 (Fig. 2), which belongs to an uncharacterized genus (QPIN01) within *Methylomonadaceae* and was recovered from cold seep sediment in the ocean. Genes encoding N_2_O reductases were exclusively found in alphaproteobacterial methanotrophs as well as in one representative genome in each of the phyla *Verrucomicrobiota* and *Gemmatimonadota* [2]. Here, we report the identification of a Clade II *nosZ* gene in a gammaproteobacterial methanotroph. Of these MAGs, 26 contained either a *nirK* or *nirS* gene and 17 harbored a *qnor* or *cnor* gene, suggesting their potential to utilize NO_2_^−^ or NO for N_2_O production. Without the addition of external NO_2_^−^, 14 strains of *Methylomonas methanica*, *Methylomonas koyamae*, and *Methylomonas lenta* have been shown to be capable of converting NH_3_ to N_2_O [14]. The coupling of aerobic CH_4_ oxidation with denitrification has been observed in rice paddy fields [60]. Recently, it has been reported that acidophilic aerobic methanotrophs can couple CH_4_ oxidation with N_2_O respiration under suboxic conditions [2]. Altogether, it is conceivable that for these aerobic methanotrophs harboring *hao* genes in NH_3_-rich marine habitats, NH_3_ oxidation could act as an alternative source of NO or NO_2_^−^, thereby contributing to N_2_O production. Furthermore, the coupling of CH_4_ oxidation and denitrification may further enhance the contribution of marine methanotrophs to N_2_O emissions.

### Evidence that marine methanotrophs contribute to N_2_O production via NH_3_ oxidation

To confirm contributions of MOB to NH_3_ oxidation and N_2_O production in estuarine ecosystems, we cultivated microbes in surface water and sediment samples from two distinct estuarine sites (ST1 and ST2) under oxic condition. The growth of MOB populations was facilitated by injecting CH_4_ into the headspace of serum bottles. Canonical NH_3_ oxidizers were removed through successive transfers into NH_3_-free medium combined with the application of specific inhibitors. After 60 days of incubation, high-throughput sequencing of 16S rRNA genes revealed that the percentage of MOB in the microbial community exhibited a substantial increase, rising from 0.2%–1.9% to 18.8%–32.7% in sediment enrichments, and from 0.01% to 23.2%–38.6% in water enrichments (Supplementary Fig. 2), compared to their initial abundances in the field samples. The 16S rRNA gene library analysis, gene quantification, and cloning of *amoA* genes revealed the absence of AOA, AOB, and comammox *Nitrospira* in the final enriched cultures (Supplementary Figs 2 and 3).

**Figure 3:**
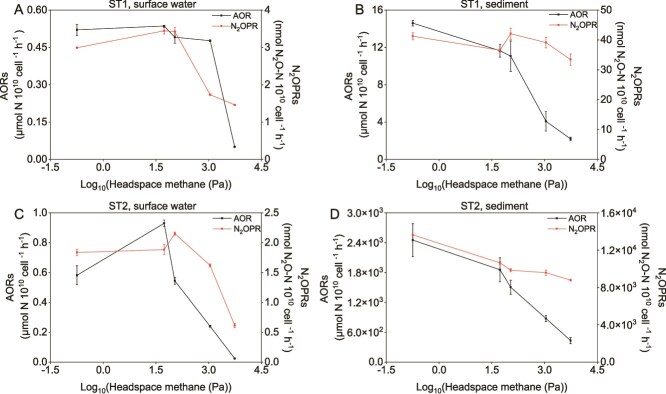
Patterns of NH_3_ oxidation rates (AORs) and N_2_O production rates (N_2_OPRs) by MOB in water (A and C) or sediment (B and D) enrichments across a gradient of CH_4_ partial pressures in the headspace of serum bottles. In the enrichments, the *amoA* gene of canonical NH_3_ oxidizers was no longer detectable, thus ^15^N-derived AORs or N_2_OPRs were considered to be exclusively mediated by MOB. Four CH_4_ concentration gradients were established in the serum bottle headspaces: 0.05% (54 Pa), 0.1% (109 Pa), 1% (1099 Pa), and 5% (5495 Pa) (v/v). The methane partial pressure is plotted on a log_10_ scale. The error bars represent the standard deviation (SD) of the mean (*n* = 3).

Using these MOB enrichments, we measured their AORs and associated N_2_OPRs under varying CH_4_ partial pressures. Under a CH_4_ pressure of 0.18 Pa (corresponding to the CH_4_ concentration in the modern atmosphere), the AORs and N_2_OPRs by MOB in the ST1 water enrichment (0‰ salinity) were 0.52 μmol N 10^10^ cell^−1^ h^−1^ and 3 nmol N_2_O–N 10^10^ cell^−1^ h^−1^, respectively (Fig. 3). In contrast, the AORs were slightly higher (0.58 μmol N 10^10^ cell^−1^ h^−1^) in the high-salinity ST2 water enrichment (10‰ salinity). Whether at the ST1 or ST2 station, the AORs and N_2_OPRs by MOB from sediment samples were 1–4 orders of magnitude higher than those by MOB from surface water samples (Fig. 3). This may result from MOB in the sediment having adapted to a long-term high-NH_3_ environment. Moreover, as the partial pressure of CH_4_ in the headspace increased, the AORs by MOB decreased rapidly, suggesting a competitive relationship between CH_4_ and NH_3_. This supports the inference that NH_3_ oxidation in the enrichments is mediated by MOB. A similar trend was observed for N_2_OPRs in both water and sediment enrichments, but the decrease in N_2_OPRs is less pronounced in sediment enrichments compared with water enrichments (Fig. 3B and D). This suggests that some of the N_2_O in sediment enrichments may originate from denitrification by MOB, which is consistent with the wide distribution of denitrification genes in MAGs recovered from diverse sediment samples. Under standard temperature and pressure conditions (e.g. 20°C), the concentration of CH_4_ in surface seawater is typically between 10 nM and 50 nM. In the Yangtze river estuary, the maximum CH_4_ concentration in the water column is around 79 nM [18]. These values are much lower than the CH_4_ concentration of 460 nM observed in the medium, which was maintained under a CH_4_ pressure of 54 Pa in the headspace of serum bottle, likely implicating that the presence of CH_4_ gas in coastal waters has a negligible inhibitory effect on NH_3_ oxidation by MOB.

**Figure 4:**
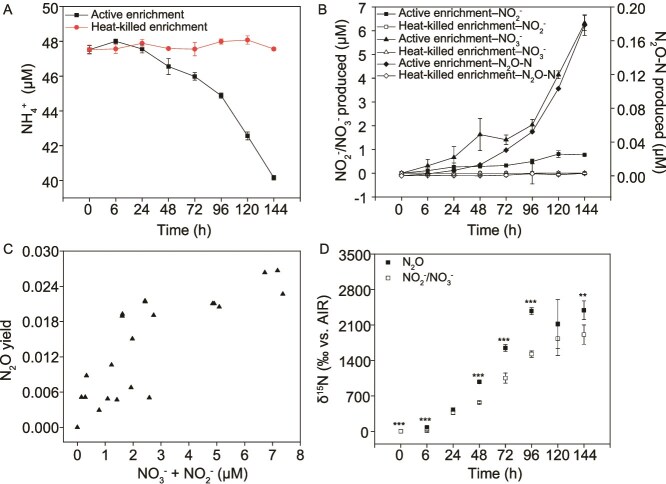
NH_3_ oxidation and N_2_O production by the MOB enrichment obtained from the ST2 water samples. (A) Time-course of NH_3_ oxidation by the MOB enrichment, (B) time-course of N_2_O, NO_2_^−^, and NO_3_^−^ production during NH_3_ oxidation by the ST2 MOB enrichment, (C) with increasing combined concentrations of NO_2_^−^ and NO_3_^−^, the N_2_O yield in MOB enrichments increased. The N_2_O yield is defined as mole of N_2_O–N produced per mole of total NO_2_^−^ and NO_3_^−^ generated. (D) Time-course of δ^15^N of N_2_O and NO_2_^−^/NO_3_^−^ produced after the addition of ^15^NH_4_Cl to MOB enrichments. The error bars represent the standard deviation (SD) of the mean (*n* = 3). The differences between N_2_O and NO_3_^−^/NO_2_^−^ were tested by two-tailed paired t-tests and labeled with ^***^ when *P* < .001, or ^**^ when *P* < .01.

Given that MOB do not use NH_3_ as an energy source [61], we next examined the duration of NH_3_ oxidation by MOB using the ST2 water enrichment where MOB accounted for 23.2% of the total microbial community. During 144 h of incubation, NH_3_ was oxidized to NO_3_^−^ in near stoichiometric amounts, accompanied by N_2_O production (Fig. 4A and B). A slight increase in NO_2_^−^ concentration was observed, suggesting that most of the NO_2_^−^ produced by MOB was subsequently converted to NO_3_^−^ by NO_2_^—^oxidizing bacteria within the enrichment. The N_2_O yield increased as the final concentration of [NO_2_^−^ + NO_3_^−^] increased (Fig. 4C), as shown for marine NH_3_ oxidizers [62, 63]. Time-course experiments with supplemented ^15^NH_4_^+^ [δ^15^N_NH4_ = 26 680‰ relative to atmospheric N_2_] revealed that the δ^15^N values in the produced N_2_O (977–2391‰) were significantly higher (two-tailed paired t-test, *P* < .01 or .001) than those in NO_2_^−^/NO_3_^−^ (569–1910‰) at all time points except 24 and 120 h after the addition of ^15^NH_4_Cl (Fig. 4D). This suggests that at least part of the N_2_O was directly derived from the ^15^NH_4_^+^ pool rather than the NO_2_^−^/NO_3_^−^ pool.

**Table 1:** Natural isotopic signatures of N_2_O produced by the MOB enrichment cultures.

	δ^15^N^α^ (‰)	δ^15^N^β^ (‰)	*SP* (‰)	δ^15^N^bulk^_N2O_ (‰)	δ^18^O_N2O_ (‰)
ST1 sediment	0.56 (0.56)	−11.27 (0.59)	11.83 (1.15)	−5.35 (0.02)	34.96 (0.81)
ST1 water	−15.42 (0.49)	−37.41 (1.56)	21.99 (2.05)	−26.41 (0.54)	17.58 (0.28)
ST2 sediment	8.39 (0.18)	−5.13 (0.35)	13.52 (0.49)	1.63 (0.13)	42.12 (1.5)
ST2 water	−12.45 (0.46)	−28.72 (0.7)	16.27 (0.54)	−20.58 (0.53)	20.79 (0.26)

Values in parentheses represent standard deviations.

The intramolecular distribution of ^15^N within the N_2_O molecule, expressed as site preference (*SP* = δ^15^N^α^–δ^15^N^β^), provides valuable insight into the source of N_2_O [35, 62, 64]. An *SP* value of ~30‰ has been associated with the formation of N_2_O through processes involving NH_2_OH oxidation of NH_3_ oxidizers, fungal denitrification, or abiotic reactions involving NH_2_OH [62, 65]. In contrast, bacterial heterotrophic denitrification or nitrifier denitrification typically result in N_2_O with an *SP* value near or below zero [62, 65]. We observed *SP* values ranging from 11.83 ± 1.15‰ to 21.99 ± 2.05‰ for N_2_O produced by these MOB enrichment cultures (Table 1), indicating that denitrification is not the primary source of N_2_O in these cultures. Instead, the *SP* values likely reflect a combination of N_2_O production from both NH_2_OH oxidation and denitrification pathways.

To provide additional evidence that NH_2_OH oxidation of MOB in these enrichments contributes to N_2_O production, we conducted metagenomic sequencing of the ST2 water enrichment, along with transcriptomic analysis before and after NH_3_ addition. From the metagenome, we recovered four medium-quality MOB MAGs (bins 35, 133, 99, and 132), two of which contained both *pmo* and *hao* genes (Supplementary Table 8). Among them, bin132, classified as a new species within the genus *Methylotuvimicrobium* (family *Methylomonadaceae*), had the highest relative abundance (13.2%) (Supplementary Table 8). Consistently, 16S rRNA library analysis of the ST2 water enrichment also indicated the dominance of *Methylotuvimicrobium* among the MOB population (Supplementary Fig. 2). These results suggest that bin132 may play a key role in NH_3_ oxidation within the ST2 water enrichment. Thus, bin132 was selected as the reference genome for metatranscriptomic analysis.

**Table 2:** Transcripts abundances of key genes involved in NH_3_ oxidation, N_2_O production, and CH_4_ metabolism in MOB enrichment cultures at 0 h, 12 h, and 24 h after NH_4_^+^ addition. Transcripts were mapped to the ST2_W_bin.132 (*Methylotuvimicrobium* sp.) reference genome reconstructed from ST2 water MOB enrichment metagenome. Negative values represent a reduction, calculated as the ratio relative to 0 h/12 h or 0 h/24 h.

Function	Gene product	Gene	Ratio, 12 h/0 h	Ratio, 24 h/0 h
NH_3_ oxidation	Methane monooxygenase subunit A	*pmoA*	2.22^**^	2.19^*^
	Methane monooxygenase subunit B	*pmoB*	2.76^**^	2.58^*^
	Methane monooxygenase subunit C	*pmoC*	2.76^*^	2.77^*^
NH_2_OH oxidation	Hydroxylamine oxidoreductase	*haoA*	2.67^**^	3.34^**^
	Hydroxylamine oxidation protein HaoB	*haoB*	2.54^*^	3.00^**^
	Cytochrome P460	*cytL*	3.67^**^	4.26^**^
NO reduction	Cytochrome c NO reductase b subunit	*cnorB*	1.65^*^	1.25^*^
	Cytochrome c NO reductase cytochrome c subunit	*cnorC*	1.78^*^	1.89
CH_3_OH oxidation	Methanol oxidation system protein MoxJ	*moxJ*	1.59	−1.12^*^
	Methanol dehydrogenase [cytochrome c] subunit	*moxF*	1.06	−1.29
CH_2_O oxidation	Formaldehyde-activating enzyme	*fae*	1.08	−1.13
H_4_MPT/H_4_F	NADP-dependent methylenetetrahydromethanopterin / methylenetetrahydrofolate dehydrogenase	*mtdA*	1.14	1.05
	NAD(P)-dependent methylenetetrahydromethanopterin dehydrogenase	*mtdB*	1.28	1.16
	Methenyltetrahydrofolate cyclohydrolase	*fchA*	−1.01	−1.06
	Formate—tetrahydrofolate ligase	*fhs*	1.58	1.56
Serine cycle	Serine hydroxymethyltransferase	*glyA*	1.2	1.08
	Enolase C-terminal domain-like protein	*eno*	−1.57	−2.22
	Malate dehydrogenase	*mdh*	1.57	1.45

Statistical significance is indicated as follows: ^*^P < .05 and ^**^P < .01

**Figure 5:**
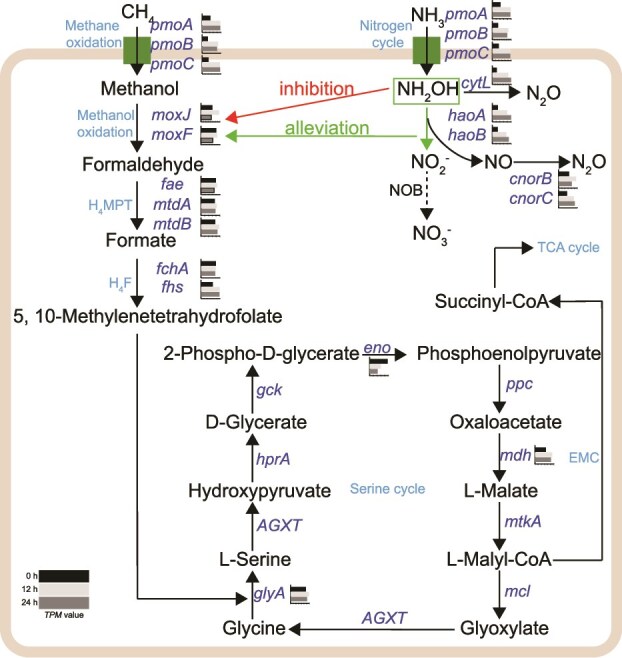
Schematic illustration of the roles of mHAO and pMMO in CH_4_ and nitrogen metabolism following NH_4_^+^ addition. The genes shown in the figure are listed in Supplementary Table 9. A horizontal clustered bar chart illustrates the relative expression levels (*TPM*) of each gene at three time points (0, 12, and 24 h). Detailed expression values are provided in Supplementary Table 9, and the bar chart legend is in the lower-left corner of the figure. NOB, nitrite-oxidizing bacteria.

We used Kallisto [54] to estimate gene expression levels by mapping metatranscriptomic reads to genes of bin132. Transcripts encoding pMMO were significantly upregulated by 2.22- to 2.76-fold at 12 h, and 2.19- to 2.77-fold at 24 h after NH_4_Cl addition (two-tailed paired t-test, *P* < .05 or .01), supporting the involvement of this enzyme in NH_3_ oxidation (Table 2 and Supplementary Table 9). Transcripts related to NH_2_OH oxidation (*hao* and *cytL*) and NO reduction (*cnorBC*) were also markedly upregulated (*P* < .05, .01, or .001) in response to NH_3_ addition, indicating their roles in N_2_O production. In contrast, no significant changes—or, in some cases, significant downregulation—were observed in transcripts encoding enzymes involved in methanol or formaldehyde oxidation, the tetrahydromethanopterin/tetrahydrofolate pathway, and the serine cycle (Table 2 and Fig. 5). These findings suggest that MOB do not couple NH_3_ oxidation with energy conservation or carbon fixation (Table 2). It is assumed that, under nutrient-limited conditions, cells downregulate the expression of nonessential genes to conserve resources and sustain basic cellular functions.

Here, we show that mHAO-containing aerobic methanotrophs are widely distributed across diverse marine habitats, with higher abundances in cold seep and estuary regions than in other environments. In addition, we identified a second order within *Gammaproteobacteria* (Ga0077536) potentially capable of aerobic methanotrophy, suggesting that unknown diversity of aerobic methanotrophs remains to be explored. NH_3_ oxidation and N_2_O production were detected in MOB enrichments from estuarine water and sediments, where canonical NH_3_ oxidizers are absent. NH_3_ oxidation by MOB can persist for up to ~6 days, and the N_2_O produced is at least partially derived from the NH_2_OH oxidation by MOB. Denitrification genes were identified in most of genomes of marine methanotrophs analyzed. It is likely that both NH_3_ or CH_4_ oxidation in methanotrophs can be coupled with denitrification to produce N_2_O in hypoxic or anoxic habitats such as marine sediment, marine aggregates, or zooplankton feces. The link between carbon and nitrogen cycles in aerobic methanotrophs may be a consequence of the gradual increase of O_2_ levels in the ancient ocean. Given the vast volume of the ocean, aerobic methanotrophs may be a significant yet underappreciated source of marine N_2_O, which partially offsets CH_4_ sequestration by themselves.

## Supplementary Material

Supplementary_material_wraf242

Supplementary_Table_wraf242

## Data Availability

Raw reads of the metagenomic and metatranscriptomic, and 16S rRNA gene sequences, as well as all MAGs, have been deposited in the NCBI database under BioProject ID PRJNA1258082. All related genomic sequences are available at Figshare (https://figshare.com/articles/dataset/MOB_genomes/30172048).
