# Supplementary material for: Ammonia oxidation by aerobic methanotrophs as a source of marine nitrous oxide
Source: ISME J. 2025 Oct 31;19(1):wraf242. doi: 10.1093/ismejo/wraf242 (PMC12636513; doi:10.1093/ismejo/wraf242)
Supplement: Supplementary_material_wraf242 [file supplementary_material_wraf242.pdf]

Supplementary Materials for

Ammonia oxidation by aerobic methanotrophs as a source of marine nitrous oxide

Sai Yang[1], Jiawei Zhang[1], Yafei Ou[1], Wenxiao Liu[1], Xinru Tian[1], Li-Jun Hou[1], Hong-Po Dong[1]*

[1]State Key Laboratory of Estuarine and Coastal Research, East China Normal University, Shanghai 200241, China

* Corresponding author:
Prof. Hong-Po Dong
State Key Laboratory of Estuarine and Coastal Research, East China Normal University, 500 Dongchuan Road, Shanghai 200241, China.
Email: hpdong@sklec.ecnu.edu.cn. Tel.: 086021-54836003

Running Title: Methanotrophs as a source of marine N_2O

Supplementary Methods

Composition of the nitrate mineral salts (NMS) medium

The NMS medium contained the following components per liter: $MgSO_4 \cdot 7H_2O$ (1.0 g), $CaCl_2 \cdot 6H_2O$ (0.20 g), KNO_3 (0.1 g), KH_2PO_4 (0.272 g), $Na_2HPO_4 \cdot 12H_2O$ (0.717 g), $CuSO_4 \cdot 5H_2O$ (0.002497 g), 2.0 mL of chelated iron solution (see below), and 0.5 mL of trace element solution. The chelated iron solution, prepared per liter, contained $FeCl_3$ (0.5 g), EDTA (2 g), and concentrated HCl (3 mL). The trace element solution, also prepared per liter, consisted of EDTA (0.5 g), $FeSO_4 \cdot 7H_2O$ (0.2 g), $ZnSO_4 \cdot 7H_2O$ (0.01 g), $MnCl_2 \cdot 4H_2O$ (0.003 g), H_3BO_3 (0.03 g), $CoCl_2 \cdot 6H_2O$ (0.02 g), $CaCl_2 \cdot 2H_2O$ (0.001 g), $NiCl_2 \cdot 6H_2O$ (0.002 g), and $Na_2MoO_4 \cdot 2H_2O$ (0.003 g).

Measurement of NH_3 oxidation and N_2O production rates

The ammonia (NH_3) oxidation rate was measured following methods outlined in previous studies [1, 2], where the $\delta^{15}N$ in NO_X^- (NO_2^- + NO_3^-) was determined with the denitrifier method. Aerobic methanotrophs (MOBs) enrichment samples were placed into 120 mL serum bottles and incubated in the dark for 24 h at 25°C with a rotation speed of 120 rpm after the addition of $^{15}NH_4Cl$ (98 atom% ^{15}N; Sigma–Aldrich; final concentration, 1 μM) and 10 μM unlabled-NH_4Cl. To terminate the reaction, all samples were centrifuged at 14,000 × g for 10 minutes. The blank control samples were centrifuged immediately after the addition of the tracer to mark the t_0 time point. The resulting supernatant was stored at -20°C until analysis. The NO_X^- in the supernatant was converted to nitrous oxide (N_2O) by denitrifiers, and then the isotopic composition of the produced N_2O was analyzed using a Delta V Advantage isotope ratio mass spectrometer (IRMS, Thermo Fisher Scientific, Bremen, Germany). The NH_3 oxidation rate (AOR) was then computed using the equations provided by previous study [3].

$$R_{nitrification} = \frac{(R_{t1}NO_X{}^{-} \times [NO_X{}^{-}]_{t1}) - (R_{t0}NO_X{}^{-} \times [NO_X{}^{-}]_{t0})}{t1 - t0} \times \frac{1}{F} \quad \text{(S1)}$$

$$F = \frac{[{}^{15}NH_4{}^{+}]}{[{}^{14}NH_4{}^{+}] + [{}^{15}NH_4{}^{+}]} \quad \text{(S2)}$$

Where, $R_{nitrification}$ represents the AOR (μmol-N L^{-1} h^{-1} or μmol-N g^{-1} *d.w.s* h^{-1}), $R_{t1}NO_X^-$ and $R_{t0}NO_X^-$ represent the ^{15}N ratio in the NO_X^- pool at times t_1 and t_0,

respectively, which is determined using the denitrification method combined with IRMS. $[NO_X^-]_{t1}$ and $[NO_X^-]_{t0}$ represent the concentration of NO_X^- at times t_1 and t_0, respectively. $[^{14}NH_4^+]$ and $[^{15}NH_4^+]$ represent the background concentration of $^{14}NH_4^+$ and the final concentration of $^{15}NH_4^+$ after adding stable isotope tracer, respectively.

The N_2O production rate (N_2OPR) was measured following our previous method with minor modifications [4]. The handling and incubation procedures for the MOB enrichment samples were consistent to those used in the NH_3 oxidation rate measurement experiment. However, two key differences were noted: 1) After adding the samples, 1 μM $^{15}NH_4Cl$ (98 atom% ^{15}N; Sigma–Aldrich) and 10 μM unlabled-NH_4Cl to the 120 mL headspace vials, the vials were immediately sealed with butyl rubber stoppers and aluminum caps, and 2) Upon completion of the reaction, saturated $ZnCl_2$ was added to kill the microorganisms and terminate the reaction. N_2O concentration in the headspace of these vials was measured using a gas chromatograph (GC-2014, Shimadzu, Kyoto, Japan). The aqueous N_2O concentration was calculated from the headspace concentrations using equation S3, with a dimensionless Henry's law constant of 2.21 for N_2O [5]. The total N_2O concentration (C_{tot}) in the incubation bottles was calculated as: $C_{tot} = C_{aq} + C_{he}$.

$$C_{aq} = \frac{C_{he}}{2.21} \times \frac{1}{24.86} \quad \text{(S3)}$$

Where, C_{aq} and C_{he} represent the aqueous and headspace concentrations (in mol L^{-1}) of N_2O, respectively.

The N_2O isotope composition was analyzed using a Delta V Advantage isotope ratio mass spectrometer (IRMS, Thermo Fisher Scientific, Bremen, Germany). Prior to sample analysis, the instrument was calibrated with N_2O reference gas pulses, which were introduced immediately before each sample elution. The $\delta^{15}N$ and $\delta^{18}O$ values of the reference gas were calibrated against international standards: atmospheric N_2 (AIR) for $\delta^{15}N$ and Vienna Standard Mean Ocean Water (VSMOW) for $\delta^{18}O$, following calibration procedures developed by S. Toyoda at Tokyo Institute of Technology[6]. The $^{15}NH_4^+$-derived N_2OPRs were calculated using the following the equation[7, 8]:

$$R_{N_2O-N} = \frac{1}{F}\left(\frac{d[^{45}N_2O]}{dt} + 2 \times \frac{d[^{46}N_2O]}{dt} \times \frac{1}{F}\right) \quad \text{(S4)}$$

In Eq. (S4), [$^{45}N_2O$] and [$^{46}N_2O$] represent concentrations of the single- and double-labeled N_2O produced during incubation, respectively. F is the fraction of ^{15}N in the NH_4^+ pool (equation S2).

Quantitative PCR

Quantitative PCR (qPCR) assays were employed to analyze the abundance of 16S rRNA genes for total bacteria and archaea, *amoA* genes for AOA, AOB, and Comammox *Nitrospira*, and *pmoA* gene for MOB. qPCR was conducted using the SYBR Green I fluorescence method in the ABI 7500 Real-Time PCR system (Thermo Fisher, USA). The target genes and blank controls were run in triplicate. Primer sequences, reaction mixture and thermal cycling conditions are provided in Supplementary Table 5. Standard curves for qPCR were constructed by 10-fold serial dilutions of plasmids containing the corresponding target genes with known concentrations, following previously described methods [9]. The qPCR results for each primer pair met the following criteria: a single peak in the dissociation curve to ensure PCR amplification specificity, an amplification efficiency between 90-110%, and a correlation coefficient (R^2) higher than 0.98.

Sequencing of 16S rRNA gene amplicons and OTU analysis

The 16S rRNA genes from *in situ* samples and samples enriched for 60 days were amplified using a two-step PCR procedure for barcoded pyrosequencing [10, 11]. Briefly, in the first step, the 16S rNRA gene was amplified for 20 cycles using 515F (with a 6-nucleotide barcode at the 5' end) and 806R primers [12, 13] (Supplementary Table 5). In the second step, the first PCR products were amplified for 5 cycles using the same primers, with an index added to the 5' end of the 806R primer [12, 13]. The library size was determined through 2% agarose gel electrophoresis, and the library concentration was quantified using the Qubit 3.0 fluorescence quantification system. The target gene bands were then recovered and purified, followed by sequencing on a Hiseq 2000 sequencer in Shanghai Biotechnology Corporation. Adapters at the 3' ends of reads 1 and reads 2 were

removed using cutadapt (v1.18) [14]. The reads were subsequently merged using the PEAR software (v0.9.8) [15]. Finally, the PRINSEQ tool (v0.20.4) was employed to trim bases from the 3' end of reads with quality values below 20 [16]. Sequencing of the 16S rRNA genes from eight samples yielded a total of 782,013 reads after quality control.

To eliminate non-target gene sequences, 16S rRNA gene sequences were filtered based on the SILVA rRNA database (v138) to remove non-bacterial and non-archaeal sequences [17]. The remaining sequences were clustered into operational taxonomic units (OTUs) using the Usearch (v11.0.667) [18, 19], based on a 97% similarity cutoff. Chimeric sequences were identified and removed using UCHIME (v4.2.40) [20]. The most abundant representative sequence of each OTU was searched against 16S rRNA sequences in NCBI database using BLASTN. Taxonomic information for hits that met the criteria (e-value < 1e-5, identity > 90%, and alignment length > 90%) was retrieved.

Metagenomic and metatranscriptomic analyses of the enrichment cultures

MOB enrichment cultures derived from ST2 water samples were centrifuged at 12,000 × g for 10 minutes at 4°C. The resulting pellets were then washed once with NMS media without nitrate (NO_3^-) and centrifuged again. A portion of the pellets was used for metagenomic sequencing. Genomic DNA was extracted using a commercial DNA extraction kit (MoBio Laboratories, Carlsbad, USA). Metagenomic sequencing was performed on the HiSeq 2500 System (Illumina) at Novogene Co., Ltd (Beijing, China), generating approximately 40 Gbp of paired-end reads (2 × 150 bp) for the enrichment sample.

Genome assembly and binning of the metagenomic data were performed as described in the main text. From the ST2 water MOB enrichment metagenome, four MOB MAGs -bin35, bin133, bin99, and bin132- were recovered (Supplementary Table 8). According to taxonomic classification using the Genome Taxonomy Database Toolkit (GTDB-Tk) [21], these MAGs were affiliated with the genera *Methylocystis*, *Methylobacter*, *Methylomonas*, and *Methylotuvimicrobium,* respectively. Among the four MAGs, two contained both *pmo* and *hao* genes, one

contained *pmo* genes but lacked *hao*, and one contained *hao* genes but lacked *pmo*. Metagenomic reads were aligned to the four MOB MAGs using Bowtie2 (v.2.3.5; --no-unal) [22]. The relative abundance of each MOB MAGs was calculated as the percentage of reads mapped to each MAG relative to the total number of clean reads. Among the four MOBs, bin132 exhibited the highest relative abundance, reaching 13.2%, suggesting that it may play a key role in NH_3 oxidation within the enrichment culture. Consequently, bin132 was selected as the reference genome for subsequent metatranscriptomic analyses.

For metatranscriptomic samples, another portion of the pellets were suspended in NMS containing NH_4Cl (1 μM $^{15}NH_4Cl$ and 10 μM non-labeled $^{14}NH_4Cl$, final concentration), excluding NO_3^-, and incubated for six days. Samples were collected at 0 h, 12 h, and 24 h for metatranscriptomic analysis. Total RNA was extracted using MagZol Reagent (R4801, Magen) following the manufacturer's instructions. rRNA, including 5S, 16S, and 23S rRNA, was removed using the Ribo-clean rRNA Depletion Kit Mega (Vazyme, RN417). cDNA libraries were then constructed using the VAHTS Universal V8 RNA-seq Library Prep Kit (Vazyme, NR605). Sequencing was performed on the NovaSeq System (Illumina) at GENWIZ (Suzhou, China), generating 10 Gbp of raw data per sample (2 × 150 bp).

Quality control of raw reads was performed using Cutadapt (v1.18) [14]. Gene sequences in the bin132 recovered from ST2 water MOB enrichment metagenome were incorporated to construct an index, and metatranscriptomic clean reads were mapped to genes of bin132 using Kallisto (v0.46.0) with default parameters to estimate transcript abundance [23]. Transcripts Per Million (*TPM*) was used to estimate the gene expression levels in a single genome.

$$TPM_i = \frac{\frac{R_i}{L_i}}{\sum_{j=1}^{n}\frac{R_j}{L_j}} \times 10^6 \qquad \text{(S5)}$$

Where, i represents a specific gene, R_i refers to the number of reads mapped to gene i, and L_i represents the length of the gene i (kb). j represents different genes, and n is the total number of genes in a genome.

Site preference (*SP*) analysis of N_2O produced from MOB enrichments

To identify the sources of N_2O production in the enrichment cultures, we performed natural isotopic analysis of N_2O. Briefly, 20 mL of the MOB enrichment cultures were transferred to 120 mL serum bottles and aerated using high-purity air. The cultures were supplemented with non-labeled $^{14}NH_4Cl$ (final concentration: 10 μM) before being sealed with butyl rubber stoppers and aluminum caps. Following a 48-h incubation period, headspace gas was collected and analyzed using isotope ratio mass spectrometry (IRMS, Delta V Plus, Thermo Fisher Scientific, Bremen, Germany). The calculation method for N_2O isotopomer ratios is as follows:

$$\delta^{15}N^{i} = \{(^{15}R^{i}_{sample} - {}^{15}R^{i}_{std})/^{15}R^{i}_{std}\} \times 1000\ (i = \alpha, \beta, bulk) \quad \text{(S6)}$$

$$\delta^{18}O = \{(^{18}R_{sample} - {}^{18}R_{std})/^{18}R_{std}\} \times 1000 \quad \text{(S7)}$$

where $^{15}R^{\alpha}$ and $^{15}R^{\beta}$ denote isotopic ratios ($^{15}N/^{14}N$) at the α (central) and β (terminal) nitrogen positions of the linear N_2O molecule, respectively. $^{15}R^{bulk}$ represents the overall $^{15}N/^{14}N$ ratio of the entire N_2O molecule, while ^{18}R denotes the $^{18}O/^{16}O$ ratio of the oxygen atom in N_2O. Std refers to standard atmospheric N_2 for nitrogen (N) or Vienna Standard Mean Ocean Water for oxygen (O). The analytical precision of the instrument was: $\delta^{15}N^{\alpha}$ better than 0.9‰, $\delta^{15}N^{bulk}$ better than 0.5‰, and $\delta^{18}O$ better than 0.6‰ [24]. The calculation methods for $\delta^{15}N^{\beta}$ and site preference (*SP*) are as follows:

$$\delta^{15}N^{\beta} = \delta^{15}N^{bulk} \times 2 - \delta^{15}N^{\alpha} \quad \text{(S8)}$$

$$SP = \delta^{15}N^{\alpha} - \delta^{15}N^{\beta} \quad \text{(S9)}$$

^{15}N-labeling experiments

After washing the MOB-enriched cultures in the same way as described for the transcriptomic analysis, time-course experiments were conducted by adding $^{15}NH_4^+$ to the culture medium. Unlabeled NH_4Cl was added to the MOB enrichments to a final concentration of 10 μM, along with 1 μM $^{15}NH_4Cl$ (98 atom% ^{15}N; Sigma–Aldrich), resulting in an initial $\delta^{15}N_{NH4}$ of approximately 26,680‰. All experimental bottles were placed in the incubator and shaken at 25°C for six days. NH_3 oxidation reaction in these bottles was terminated by adding 100 μL saturated $ZnCl_2$ at time zero and

each of seven additional time points. The $^{15}N/^{14}N$ ratio of N_2O in the headspace was determined using Isotope Ratio Mass Spectrometry (IRMS, Delta V Plus, Thermo Fisher Scientific, Bremen, Germany). The $^{15}N/^{14}N$ ratios of NO_2^- and NO_3^- generated in the liquid were measured using a denitrifier method. $\delta^{15}N_{N2O}$ was caculated as follws:

$$\delta^{15}N_{N_2O} = \{(R_{sample}/R_{standard}) - 1\} \times 1000 \qquad \text{(S10)}$$

where, R_{sample} represents the $^{15}N/^{14}N$ ratio in the sample, and $R_{standard}$ is the $^{15}N/^{14}N$ ratio in standard atmospheric N_2, which is 0.0036765.

Supplementary Figures

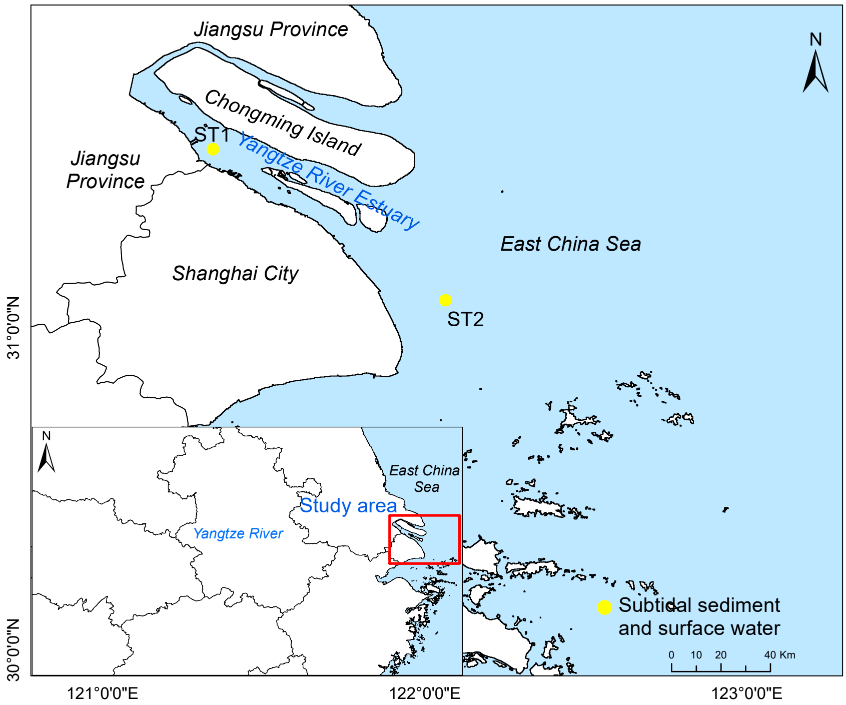

Supplementary Fig. 1 Study area and sampling sites. This figure shows the location of the Yangtze estuary and the sampling sites. ST1 and ST2, two sites ranging from the estuary mouth to its adjacent coastal area. The research area was marked by the red box.

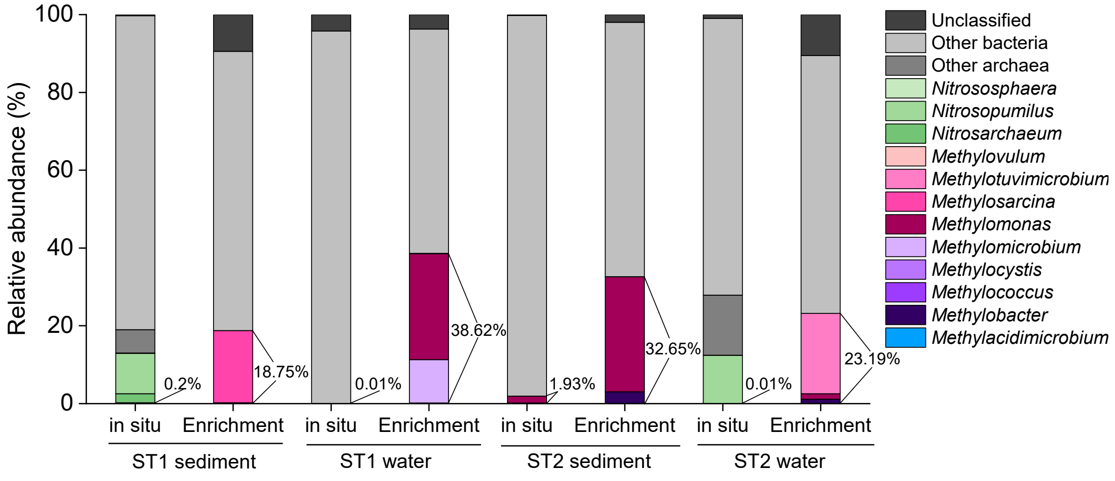

Supplementary Fig. 2 Microbial community composition of in situ samples and MOB enrichment cultures derived from sediment and surface water at sites ST1 and ST2. The 16S rRNA gene was amplified using the universal primers 515F and 806R [12, 13], which target both bacteria and archaea within the same sequencing library. The percentages displayed on the right side of each bar indicate the relative abundance of MOB within the total microbial community.

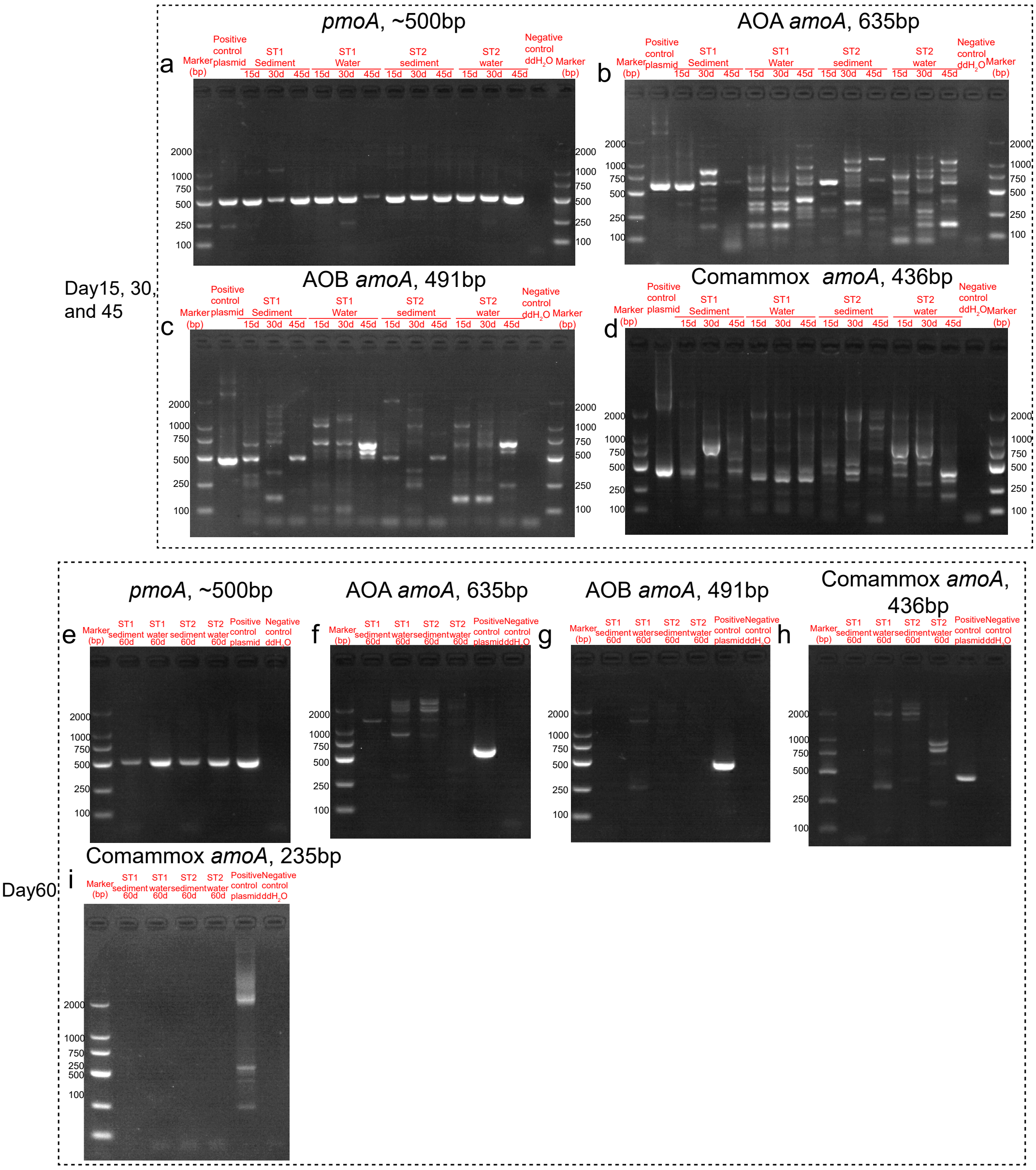

Supplementary Fig. 3 Agarose gel detection of PCR products for the *amoA* and *pmoA* genes of microorganisms during the MOB enrichment process. The positive control is a plasmid containing the target gene fragment. **a-d.** Cultures after 15, 30, and 45 days of enrichment. **e-i** Cultures after 60 days of enrichment. The primer comamoA F/R was in panel d and h. The primer CA377F and C576R was used in panel i.

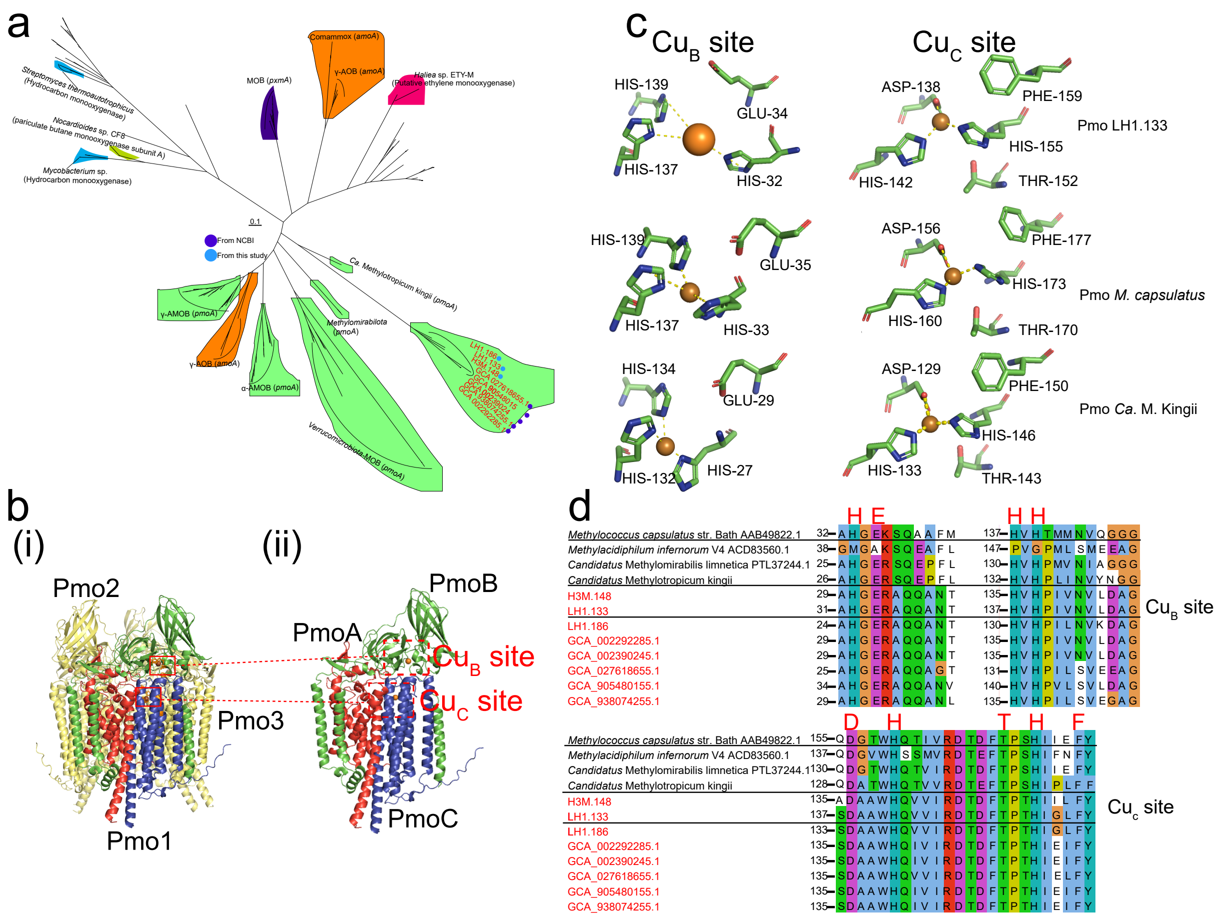

Supplementary Fig. 4 Analysis of the particulate methane monooxygenases from newly discovered aerobic methane oxidation bacteria in sampled coastal wetlands. **a**. Unrooted tree of amino acid sequences of particulate methane monooxygenase A subunit (PmoA). The putative novel PmoA sequences are highlighted in red font, with three sequences retrieved from our bins and five from publicly available genomes. The phylogenetic tree was constructed using IQ-TREE with the model LG+F+I+G4. In addition to the know PmoA sequences, the reference sequences also include members of the copper membrane monooxygenase superfamily, such as AmoA, HmoA, and PxmA. The scale bar indicates 0.1 amino acid substitutions per site. **b**. The molecular model of the putative particulate methane monooxygenase (Pmo) from the genome LH1.133. (i) The molecular model of the homotrimer Pmo complex is shown in cartoon representation. The Pmo complex, positioned in the foreground, is color-coded in red, blue, and green. (ii) An enlarged view of one Pmo complex from (i), showing the sites of Cu_B and Cu_C. **c**. Stick representation of residues at the Cu_B

and Cu_C active sites of the Pmo from LH1.133, *Ca*. Methylotropicum kingii (*Gemmatimonadota*) and *Methylococcus capsulatus* (Proteobacteria). **d**. Sequence alignment of the metal-binding sites of Cu_B and Cu_C. The alignments include Pmo sequences of members of the newly discovered MOB, as well as those from *Ca*. M. kingii, *M*. *capsulatus*, *Candidatus* Methylomirabilis limnetica, and *Methylacidiphilum infernorum* (*Verrucomicrobiota*). Sites involved in Cu coordination are marked with red letters.

Supplementary References

1. Casciotti KL, Sigman DM, Hastings MG *et al*. Measurement of the oxygen isotopic composition of nitrate in seawater and freshwater using the denitrifier method. *Anal Chem* 2002;**74**:4905-12. https://doi.org/10.1021/ac020113w

2. Sigman DM, Casciotti KL, Andreani M *et al*. A bacterial method for the nitrogen isotopic analysis of nitrate in seawater and freshwater. *Anal Chem* 2001;**73**:4145-53. https://doi.org/10.1021/ac010088e

3. Peng X, Fuchsman CA, Jayakumar A *et al*. Revisiting nitrification in the Eastern Tropical South Pacific: a focus on controls. *J Geophys Res-Oceans* 2016;**121**:1667-84. https://doi.org/10.1002/2015jc011455

4. Mao T-Q, Zhang Y, Ou Y-F *et al*. Temperature differentially regulates estuarine microbial N_2O production along a salinity gradient. *Water Res* 2024;**267**:122454. https://doi.org/10.1016/j.watres.2024.122454

5. Yoon S, Nissen S, Park D *et al*. Nitrous oxide reduction kinetics distinguish Bacteria harboring clade I NosZ from those harboring clade II NosZ. *Appl Environ Microb* 2016;**82**:3793-800. https://doi.org/10.1128/aem.00409-16

6. Mcilvin MR, Casciotti KL. Fully automated system for stable isotopic analyses of dissolved nitrous oxide at natural abundance levels. *Limnol Oceanogr-Meth* 2010;**8**:54-66. https://doi.org/10.4319/lom.2010.8.54

7. Santoro AE, Buchwald C, Knapp AN *et al*. Nitrification and nitrous oxide production in the offshore waters of the Eastern Tropical South Pacific. *Global Biogeochem Cy* 2021;**35**:e2020GB006716. https://doi.org/10.1029/2020gb006716

8. Trimmer M, Chronopoulou P-M, Maanoja ST *et al*. Nitrous oxide as a function of oxygen and archaeal gene abundance in the North Pacific. *Nat Commun* 2016;**7**:13451. https://doi.org/10.1038/ncomms13451

9. Zheng YL, Hou LJ, Newell S *et al*. Community dynamics and activity of ammonia-oxidizing prokaryotes in intertidal sediments of the Yangtze Estuary. *Appl Environ Microb* 2014;**80**:408-19. https://doi.org/10.1128/aem.03035-13

10. Jones CM, Spor A, Brennan FP *et al*. Recently identified microbial guild mediates soil N_2O sink capacity. *Nat Clim Change* 2014;**4**:801-05.

https://doi.org/10.1038/nclimate2301

11. Berry D, Ben Mahfoudh K, Wagner M *et al*. Barcoded primers used in multiplex amplicon pyrosequencing bias amplification. *Appl Environ Microb* 2011;**77**:7846-49. https://doi.org/10.1128/aem.05220-11

12. Gruber-Vodicka HR, Seah BK, Pruesse E. phyloFlash: rapid small-subunit rRNA profiling and targeted assembly from metagenomes. *Msystems* 2020;**5**:e00920-20. https://doi.org/10.1128/msystems.00920-20

13. Apprill A, Mcnally S, Parsons R *et al*. Minor revision to V4 region SSU rRNA 806R gene primer greatly increases detection of SAR11 bacterioplankton. *Aquat Microb Ecol* 2015;**75**:129-37. https://doi.org/10.3354/ame01753

14. Martin M. Cutadapt removes adapter sequences from high-throughput sequencing reads. *EMBnet. journal* 2011;**17**:10-12. https://doi.org/10.14806/ej.17.1.200

15. Zhang J, Kobert K, Flouri T *et al*. PEAR: a fast and accurate Illumina Paired-End reAd mergeR. *Bioinformatics* 2014;**30**:614-20. https://doi.org/10.1093/bioinformatics/btt593

16. Schmieder R, Edwards R. Quality control and preprocessing of metagenomic datasets. *Bioinformatics* 2011;**27**:863-64. https://doi.org/10.1093/bioinformatics/btr026

17. Pruesse E, Peplies J, Glöckner FO. SINA: Accurate high-throughput multiple sequence alignment of ribosomal RNA genes. *Bioinformatics* 2012;**28**:1823-29. https://doi.org/10.1093/bioinformatics/bts252

18. Edgar RC. UPARSE: highly accurate OTU sequences from microbial amplicon reads. *Nat Methods* 2013;**10**:996-98. https://doi.org/10.1038/nmeth.2604

19. Edgar RC. SINTAX: a simple non-Bayesian taxonomy classifier for 16S and ITS sequences. *bioRxiv* 2016:074161. https://doi.org/10.1101/074161

20. Edgar RC, Haas BJ, Clemente JC *et al*. UCHIME improves sensitivity and speed of chimera detection. *Bioinformatics* 2011;**27**:2194-200. https://doi.org/10.1093/bioinformatics/btr381

21. Chaumeil PA, Mussig AJ, Hugenholtz P *et al*. GTDB-Tk: a toolkit to classify

genomes with the Genome Taxonomy Database. *Bioinformatics* 2020;**36**:1925–27. https://doi.org/10.1093/bioinformatics/btz848

22. Langmead B, Salzberg SL. Fast gapped-read alignment with Bowtie 2. *Nat Methods* 2012;**9**:357-59. https://doi.org/10.1038/nmeth.1923

23. Bray NL, Pimentel H, Melsted P *et al*. Near-optimal probabilistic RNA-seq quantification. *Nat Biotechnol* 2016;**34**:525-27. https://doi.org/10.1038/nbt.3519

24. Gao DZ, Hou LJ, Liu M *et al*. N_2O emission dynamics along an intertidal elevation gradient in a subtropical estuary: Importance of N_2O consumption. *Environ Res* 2022;**205**:112432. https://doi.org/10.1016/j.envres.2021.112432
